# Clinical Cases and the Molecular Profiling of a Novel Childhood Encephalopathy-Causing *GNAO1* Mutation P170R

**DOI:** 10.3390/cells12202469

**Published:** 2023-10-17

**Authors:** Yonika A. Larasati, Gonzalo P. Solis, Alexey Koval, Silja T. Griffiths, Ragnhild Berentsen, Ingvild Aukrust, Gaetan Lesca, Nicolas Chatron, Dorothée Ville, Christian M. Korff, Vladimir L. Katanaev

**Affiliations:** 1 Translational Research Center in Oncohaematology, Department of Cell Physiology and Metabolism, Faculty of Medicine, University of Geneva, CH-1211 Geneva, Switzerlandgonzalo.solis@unige.ch (G.P.S.); alexey.koval@unige.ch (A.K.); 2 Department of Pediatrics, Haukeland University Hospital, 5009 Bergen, Norway; 3 Department of Medical Genetics, Haukeland University Hospital, 5009 Bergen, Norway; ragnhild.drage.berentsen@helse-bergen.no (R.B.);; 4 Department of Clinical Science, University of Bergen, 5008 Bergen, Norway; 5 Department of Medical Genetics, University Hospital of Lyon, 69002 Lyon, France; gaetan.lesca@chu-lyon.fr (G.L.); nicolas.chatron@chu-lyon.fr (N.C.); 6 Pediatric Neurology Department, University Hospital of Lyon, 69002 Lyon, France; dorothee.ville@chu-lyon.fr; 7 Pediatric Neurology Unit, University Hospitals of Geneva, CH-1211 Geneva, Switzerland; christian.korff@hcuge.ch; 8 Institute of Life Sciences and Biomedicine, Far Eastern Federal University, Vladivostok 690090, Russia

**Keywords:** pediatric encephalopathy, *GNAO1*, G proteins, Gαo, dominant mutation, case report, molecular etiology, GTP binding, protein–protein interactions, intracellular localization, personalized medicine, drug discovery

## Abstract

De novo mutations in *GNAO1*, the gene encoding the major neuronal G protein Gαo, cause a spectrum of pediatric encephalopathies with seizures, motor dysfunction, and developmental delay. Of the >80 distinct missense pathogenic variants, many appear to uniformly destabilize the guanine nucleotide handling of the mutant protein, speeding up GTP uptake and deactivating GTP hydrolysis. Zinc supplementation emerges as a promising treatment option for this disease, as Zn^2+^ ions reactivate the GTP hydrolysis on the mutant Gαo and restore cellular interactions for some of the mutants studied earlier. The molecular etiology of *GNAO1* encephalopathies needs further elucidation as a prerequisite for the development of efficient therapeutic approaches. In this work, we combine clinical and medical genetics analysis of a novel *GNAO1* mutation with an in-depth molecular dissection of the resultant protein variant. We identify two unrelated patients from Norway and France with a previously unknown mutation in *GNAO1*, c.509C>G that results in the production of the Pro170Arg mutant Gαo, leading to severe developmental and epileptic encephalopathy. Molecular investigations of Pro170Arg identify this mutant as a unique representative of the pathogenic variants. Its 100-fold-accelerated GTP uptake is not accompanied by a loss in GTP hydrolysis; Zn^2+^ ions induce a previously unseen effect on the mutant, forcing it to lose the bound GTP. Our work combining clinical and molecular analyses discovers a novel, biochemically distinct pathogenic missense variant of *GNAO1* laying the ground for personalized treatment development.

## 1. Introduction

The first mutations in the gene *GNAO1* were uncovered to cause early-onset epileptic encephalopathy (EOEE) in 2013 with the advent of next-generation sequencing [1]. Since then, a number of *GNAO1* mutations have been linked with different pathological phenotypes, primarily including epilepsy and motor development disorders. Developmental delay is also frequently experienced by patients, with variable severity [2,3]. Currently, around 80 missense variants in Gαo, the protein product of *GNAO1*, reported in the ClinVar database [4] are assigned the status of “pathogenic” or “likely pathogenic”. So far, the specific correlation between the exact mutation and the severity and specific characteristics of the disease remains unclear, with more research being necessary to improve our understanding of the disease.

*GNAO1* encodes an intracellular membrane-associated Gαo protein, the α-subunit of the heterotrimeric G protein Go [5]. The α-subunits, unlike the more promiscuous β- and γ-subunits also constituting the heterotrimer, define the selectivity in coupling to G protein-coupled receptors (GPCRs) and fall into four classes: Gαi/o, Gαs, Gαq, and Gα12/13. A ligand-activated GPCR elicits the guanine nucleotide exchange factor (GEF) activity towards the GDP-loaded Gα-subunit, resulting in heterotrimer dissociation into Gα-GTP and Gβγ [6]. The system returns to its initial state via the GTPase activity of Gα, which can be further stimulated using specialized RGS (regulator of G-protein signaling) proteins, and a subsequent reassociation with Gβγ [7]. If the stimulus persists sufficiently long, this cycle can repeat multiple times until the receptor is inactivated by specific mechanisms, such as β-arrestin-mediated internalization [8].

Both the development and operation of the mammalian brain have been shown to heavily rely on Gαo, which is the major neuronal G-protein. This is illustrated by the fact that upon a full knockout of this subunit, mice exhibit developmental delays 3 weeks after birth; impairments of motor control, neurological phenotypes like hyperalgesia, hyperactivity, tremor, and seizures have been reported in these animals in adulthood [9]. More recently, cortical developmental abnormalities have also been discovered [10], which are corroborated by our own recent findings on brain development defects in mice with EOEE-causing C215Y and G203R mutations [11]. Despite these advances, the molecular and cellular mechanisms driven by Gαo are not fully clear. Consequently, the molecular etiology of *GNAO1* encephalopathies, which appear to be driven in many cases via neomorphic mutations [12,13], needs further elucidation as a prerequisite for the development of efficient therapeutic approaches.

We have previously identified salts of zinc as a potential treatment option for a subset of pathogenic Gαo variants [12]. Restoring the deficient GTPase activity of the G203R, E246K, and R209C mutants, zinc treatment also restores the aberrant cellular interactions of the mutant Gαo. In a *Drosophila* model of the disease based on the introduction of the G203R mutation into the endogenous Gαo locus that is characterized by strong motor dysfunction, reduced lifespan, and partial brain degeneration, we have found that dietary zinc supplementation partially rescues the disease phenotypes [12,14]. As oral zinc supplementation is an approved treatment for a multitude of disorders, including Wilson disease and various neurological conditions [15,16], our discovery may have identified the first causal treatment option for *GNAO1* encephalopathies.

This current study describes a novel *GNAO1* mutation c.509C>G that results in the substitution of Pro170 to Arg (P170R) in two unrelated patients from Norway and France, providing a detailed clinical description and a biochemical and cellular characterization of this mutation. In contrast to many other *GNAO1* mutations described earlier, this epilepsy- and motor dysfunction-causing protein change demonstrates the preservation of the GTPase activity in the context of an extremely fast GTP uptake. Further, in the cellular milieu, the mutant protein exhibits a poor ability to form the heterotrimer and to interact with RGS19, as well as an aberrant intracellular localization. While Zn^2+^ salts have been found to correct the abnormal behavior of some pathogenic *GNAO1* mutations at the biochemical, cellular, and animal model levels [12,17], we here further report an unusual response of Gαo[P170R] to Zn^2+^, identifying this mutant as a novel subtype of *GNAO1* mutations. Altogether, this work may represent an important milestone in the molecular and clinical elucidation of some of the severe developmental and epileptic encephalopathies.

## 2. Materials and Methods

### 2.1. Ethics Statement

Written informed consent was obtained from parents for genetic testing and for publication of the case report.

### 2.2. Genetic Investigation

DNA was isolated from the peripheral blood. High-density single nucleotide polymorphism (SNP) array Cytoscan HD (Thermo Fischer Scientific, Waltham, MA, USA) was performed to search for copy number variations (CNVs) and shared long contiguous stretches of homozygosity (LCSH) > 5 Mb. Karyotyping (G-banding) was performed on metaphase chromosomes cultured from lymphocyte cells. The chromosomes were stained with Leishman’s stain.

In the first proband, a targeted panel of 2437 genes associated with intellectual disabilities was performed and extracted from whole exome sequencing (WES) data. WES was performed for the proband and both the parents (trio). Exome enrichment and sequencing were carried out using the Roche KAPA HyperCapture Reagent Kit and Illumina NovaSeq 6000 (Illumina, San Diego, CA, USA). Average coverage was approximately 112× for the proband. The specific variant in *GNAO1* was covered by 105 reads in the proband. The variant was verified by Sanger sequencing in the proband. The variant was interpreted using the guidelines from American College of Medical Genetics and Genomics (ACMG) [18]. For the second proband, the 150-gene panel for epileptic disorders using the SeqCap EZ Roche technology for library building and a NextSeq500 for sequencing was performed.

### 2.3. Antibodies

The mouse monoclonal antibody (mAb) against mRFP (sc-101526) was from Santa Cruz Biotechnology, the mouse mAb against GM130 was from BD Transduction Laboratories (610822), and the mAb against α-tubulin was from Sigma-Aldrich (T6199). The rat mAb against HA (11867423001) was from Roche. The rabbit polyclonal antibody (pAb) against GFP (GTX113617) was from GeneTex. Peroxidase-conjugated secondary Abs for Western blots (115-035-146, 111-035-144, and 112-035-143) and the Alexa Fluor 594-labeled secondary Ab for immunostaining (115-585-146) were from Jackson ImmunoResearch.

### 2.4. Plasmids and Molecular Cloning

The plasmids for Gαo-GFP wild-type and Q205L (GFP sequence inserted between residues Gly92 and Ile93 of the Gαo), mRFP-Gβ1, and mRFP-Gγ3 were previously described [19]. For the generation of the 3xHA-RGS19 construct, the RGS19 sequence was cut with BamHI and PspOMI from the 6xHis_6_-RGS19 plasmid [20] and ligated in frame into the BglII/PspOMI sites of the p3xHA-C1 plasmid [21]. To introduce the P170R mutation, we have employed a site-directed mutagenesis approach on the following plasmids: pET23b-Gαo for bacterial expression of the N-terminally His_6_-tagged Gαo, Gαo-GFP producing Gαo internally tagged at the Gly92 position for localization and immunoprecipitation studies. In both cases, the following primers were used: 5′-CGACTACCAGCGCACCGAGCAGGACATCCTCCGAACC; 5′-GTCCTGCTCGGTGCGCTGGTAGTCGGCGGCCCCAATC. Sanger sequencing was performed on the resulting colonies to confirm the successful introduction of the substitution.

### 2.5. Fluorescence-Based Test for GTP Binding and Hydrolysis

For the GTP binding and hydrolysis assay, we employed fluorescently labeled BODIPY-GTP or BODIPY-GTPγS (Invitrogen, Waltham, MA, USA), as described previously [17,20]. Recombinant His_6_-Gαo (purified as described in the Supplementary Materials) was diluted to 1 µM in the reaction solution (TBS supplemented with 10 mM MgCl_2_ and 0.5% BSA) and indicated compounds. After that, BODIPY-GTP or BODIPY-GTPγS was injected into the wells of 384-well black Greiner plates using an injector system of the Tecan Infinite M200 PRO plate reader to a final concentration of 1 µM. The reading was performed with excitation at 485 nm and emission at 530 nm at 28 °C. To derive the *k_bind_* and *k_hydr_* rate constants, we have applied the previously reported approach of fitting GTP binding and hydrolysis kinetic curves [12,20]. GTP binding/hydrolysis assays in the presence of ZnCl_2_ and EGTA are described in the Supplementary Materials.

### 2.6. Cell Line and Culture Conditions

The mouse neuroblastoma Neuro-2a (N2a; ATCC CCL-131) cell line was cultured in Minimum Essential Medium (MEM; Thermo Fisher Scientific), supplemented with 10% FCS, 2 mM L-glutamine, 1 mM pyruvate, and 1% penicillin-streptomycin at 37 °C and 5% CO2. All plasmid transfections were carried out with X-tremeGENE HP (Roche, Basel, Switzerland, 6366546001) or *Trans*IT-2020 (Mirus, Madison, WI, USA, MIR5400) according to the manufacturer’s instructions.

### 2.7. Co-Immunoprecipitation

The GST-tagged nanobody against GFP [22] was expressed in *Escherichia coli* Rosetta-gami (Novagen, Burlington, MA, USA) and purified with glutathione Sepharose 4B beads according to the manufacturer’s instructions. Recombinant protein purity was controlled via SDS-PAGE and Coomassie blue staining.

N2a cells were seeded in 6-well plates (4 × 10^5^ cell/well), and 24 h later, cells were transfected with the Gαo-GFP constructs and mRFP-Gβ1/mRFP-Gγ3 (1:1:1 plasmid ratio) or the HA-RGS19 construct (1:1 plasmid ratio). After 24 h of transfection, cells were resuspended in ice-cold GST lysis buffer (20 mM Tris-HCl, pH 8.0, 1% Triton X-100, and 10% glycerol in PBS) supplemented with a protease inhibitor cocktail (Roche) and passed 15 times through a 25 G needle. Extracts were centrifugated at 15,000× *g* for 15 min at 4 °C, and cleared supernatants were incubated with 2 µg of purified anti-GFP nanobody for 30 min on ice. Then, 20 µL of Glutathione Sepharose 4B beads (Cytiva) were added, and samples were rotated overnight at 4 °C. Beads were repeatedly washed with GST-lysis buffer, prepared for SDS-PAGE, and finally analyzed via Western blot using antibodies against GFP (dilution 1:2000), mRFP (dilution 1:200), and/or HA-tag (dilution 1:2000), as well as HRP-conjugated secondary antibodies for ECL detection. Quantification of blots was done using ImageJ.

### 2.8. Molecular Modeling

Molecular modeling of the mutant protein was performed with default settings using the SWISS MODEL server [23]. Analysis and visualization of the resulting structure were performed in PyMol.

### 2.9. Determination of Cellular Stability of Gαo

N2a cells (2 × 10^5^ cells) were seeded in 12-well plates (2 × 10^5^ cells/well). The next day, cells were transfected with plasmids encoding Gαo-GFP or Gαo[P170R]-GFP (internally tagged GFP). After 24 h, cells were treated with cycloheximide (300 µM) before being harvested at the indicated time points (1, 3, 6, and 9 h). Cells were harvested with ice-cold lysis buffer (20 mM Tris-HCl (pH 8.0), 1% Triton X-100, 10% glycerol in PBS, 0.1% SDS, 1 mM DTT) supplemented with a protease inhibitor cocktail (Roche). Cell lysates were cleared via centrifugation at 16,000× *g* for 15 min at 4 °C and boiled with the SDS-PAGE sample buffer. Samples were lastly analyzed via SDS-PAGE, followed by Western blot using antibodies against GFP (dilution 1:2000) and β-actin (dilution, 1:2000; Proteintech, 81115-1-RR). Quantification of blots was conducted using ImageJ (version 1.53t). 

### 2.10. Immunofluorescence and Microscopy

N2a cells were seeded in 12-well plates (2 × 10^5^ cells/well) and, 24 h later, were transfected for 6 h, trypsinized, and seeded on poly-L-lysine-coated coverslips in complete MEM for 15 h before fixation. Cells were fixed for 20 min with a solution of 4% paraformaldehyde in PBS. Cells were then permeabilized for 1 min using an ice-cold solution of 0.1% Triton X-100 in PBS, blocked for 30 min with 1% BSA in PBS, incubated with the primary antibody in blocking buffer for 2 h at room temperature (RT), washed with PBS, and subsequently incubated with an Alexa Flour 594-conjugated secondary antibody and DAPI in blocking solution for 2 h at RT. Coverslips were finally mounted with Vectashield (Vector Laboratories; H-1700) on microscope slides. Cells were recorded with a Plan-Apochromat 63×/1.4 oil objective on an LSM700 Confocal Microscope and further processed using the Zeiss ZEN 3.3 (blue edition) software (all Carl Zeiss, Jena, Germany).

## 3. Results

### 3.1. Early Onset Encephalopathy and a Developmental Delay Are Driven by c.509C>G Substitution in GNAO1 Gene

**Figure 1:**
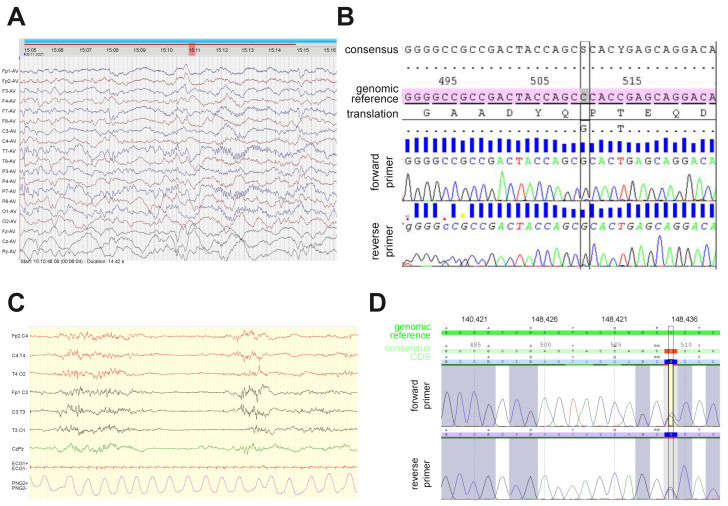
Electroencephalogram (EEG) recordings and Sanger re-sequencing in two patients with the novel c.509C>G (Pro170Arg) *GNAO1* mutation. (**A**) Interictal EEG in the Norwegian patient recorded at 10 weeks after birth showed left hemisphere abnormalities. (**B**) Results of Sanger re-sequencing of the PCR-amplified fragment of exon 5 in the Norwegian patient’s *GNAO1* allele confirm the presence of heterozygous c.509C>G substitution that results in the P170R amino acid mutation. Reference alleles with genomic numbering and consensus with CDS numbering are used for comparison. (**C**) The first EEG of the French patient showed a discontinuous pattern with multifocal spikes. (**D**) Sanger re-sequencing in the French patient confirms the heterozygous c.509C>G substitution.

The first proband is a young girl, delivered at term after a normal pregnancy to healthy non-consanguineous parents of Norwegian (father) and German (mother) origin, who already had two older healthy common children. Her birth weight was 3825 g, length was 50 cm, and head circumference measured at 1 week was 34.2 cm (−1.21 SD). At 10 weeks, the patient presented with paroxysmal events identified as electroclinical epileptic spasms. The interictal EEG showed focal spikes mainly on the left side, bordering on non-convulsive status epilepticus (Figure 1A). The brain MRI was normal, and metabolic screening tests in urine, plasma, and spinal fluid were within the normal range. The administration of phenobarbital, replaced with vigabatrin (up to 100 mg BID) rapidly improved her condition and allowed the complete seizure control. Levetiracetam was used in place of vigabatrin at 6 months due to the parents’ concerns about stomach aches and the risk of visual field constriction. This treatment is currently administered at the dosage of 17.5 mg/kg/day and has proven to be efficient, as no seizures have been observed since its introduction.

The parents observed their daughter’s slow but steady developmental progress up to 16 months (her current age), and they determined that her global abilities were equivalent to those of a child aged between 6 and 8 months. She is hypotonic, unable to sit without support, and communicates only by a few sounds and babbles. She has good eye contact, appears to be attentive to her environment, grasps objects presented to her, and rolls over from back to belly. Her management includes physical therapy and integrated kindergarten.

Trio-based WES revealed the presence of a heterozygous de novo variant in *GNAO1* (NM_020988.3) c.509C>G p.(Pro170Arg). The variant was interpreted as likely pathogenic according to the ACMG guidelines (PS2, PM2, PP2, PP3). The variant was not registered in the Genome Aggregation Database (gnomAD), and the involved Pro residue is highly conserved across species. The missense Z-score for *GNAO1* from gnomAD is 3.19, indicating that the gene is intolerant to missense variation [24]. The in silico prediction tool REVEL scored the variant as pathogenic (score 0.961) [25]. The girl was also investigated with an SNP array revealing a Turner syndrome mosaicism, which was also confirmed via standard G-banding showing a mos 45,X [6]/46,XX [24] karyotype in blood. At her current age of 16 months, her height is −3 SD, which may be due to her Turner syndrome mosaicism, but she does not have other clinical signs of Turner syndrome. The current head circumference is −3.6 SD. The echocardiogram and renal ultrasound are normal. Sanger sequencing was used to confirm the variant (Figure 1B), which results in the expression of the Gαo[P170R] mutant protein.

The second patient, a boy, is the fifth child of healthy non-consanguineous Guinean parents. He was born at term after a normal pregnancy (weight 3330 g, height 51 cm, head circumference 34 cm). He developed a severe early developmental and epileptic encephalopathy, with the first seizures occurring at 2 days with startles and tonic fits. The first EEG (Figure 1C) showed a discontinuous pattern with multifocal spikes; 10 subclinical focal discharges probably beginning in the occipital areas, as well as myoclonic jerks without a clear electro-clinical correlatoin, were recorded. Despite multiple antiepileptic drugs (vigabatrin, valproate, pyridoxin, carbamazepine, topiramate, lamotrigine, phenobarbital, clonazepam, ketogenic diet, levetiracetam, rufinamide, and zonisamide), his seizures recurred multiple times daily with variable semiology (mainly tonic and focal seizures, neither spasm nor myoclonia reported recently); the different EEG patterns recorded were non-specific with the slow background activity, poor organization, and multifocal spikes.

This severe epilepsy is associated with a profound encephalopathy; the neurological status was abnormal from the first days of life, with hypotonia, poor eye contact, and poor motricity and limb hypertonia; no abnormal movements were reported. On evolution, no psychomotor progress was noted, and the major axial hypotonia and limb hypertonia remained. The boy currently needs enteral feeding support with gastrostomy and has a chronic respiratory insufficiency.

Neither particular morphological features nor cardiac, renal, or hepatic impairments were reported. A large screening was performed and showed normal results, including cerebral MRI, amino acid chromatography in blood, CSF, and urine, urine organic acid chromatography, lactate/pyruvate in blood and CSF, biotinidase activity, urine alpha-aminoadipic-semialdehyde acid, array-CGH, and CSF neurotransmitters. Analysis of a panel of epilepsy genes, confirmed via Sanger sequencing (Figure 1D), showed a heterozygous variant in *GNAO1* (NM_020988.3) c.509C>G p.(Pro170Arg), the same as described above.

We identified that the two patients with the same novel mutation c.509C>G p.(Pro170Arg, abbreviated P170R below) share several clinical features (early onset epilepsy, movement dysfunction, developmental delay) yet are distinct in the severity of the epileptic manifestations. More patients with the same mutation would need to be identified prior to making interpretations of these differences. At this stage, we only wish to state that the lack of clear genotype–phenotype correlation was observed for other *GNAO1* mutations [26].

### 3.2. Biochemical Characterization of the Purified P170R Mutant Reveals Highly Accelerated GTP Uptake with a Mild Impairment of Hydrolysis

**Figure 2:**
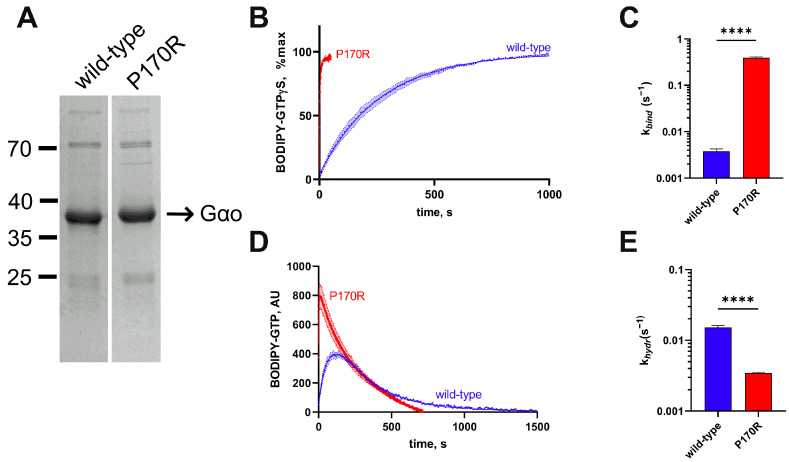
Biochemical characterization of the His_6_-Gαo[P170R] mutant protein. (**A**) Coomassie-stained gel analysis of recombinant His_6_-tagged P170R and wild-type proteins immediately after purification from *E. coli*. Both proteins were expressed in similar quantities and were of comparable purity. (**B**) The curves of BODIPY-GTPγS binding to His_6_-tagged wild-type or to the P170R mutant and (**C**) quantification of the binding rate constants (*k_bind_*) demonstrate that the mutant is around 100-fold faster in terms of BODIPY-GTPγS uptake. (**D**) BODIPY-GTP hydrolysis curves of His_6_-tagged mutant and wild-type proteins and (**E**) quantification of the hydrolysis rate constant (*k_hydr_*) show around 3-fold slower BODIPY-GTP hydrolysis in the mutant. Statistical comparison for panels (**C**,**E**) was performed by Student’s *t*-test, significance is shown as **** *p* < 0.0001.

Using the *E. coli* expression system, we produced His_6_-Gαo[P170R] and compared its kinetic properties to those of wild-type His_6_-Gαo (Figure 2A) [19]. N-terminal hexahistidine tagging of Gαo has been previously used by us and shown to produce a biochemically active protein with normal protein–protein interactions [12,13,17,19,20,21,27,28]. GTP uptake was measured using the BODIPY-GTPγS binding assay, in which we continuously tracked the accumulation of the GTP-bound form of the protein prior to fitting the resulting kinetic curve to determine the rate constant (Figure 2B), which reliably reflects the rate constant of the non-labeled GTP [20,29]. As evident from both the overall appearance of the curves in this assay and the quantification of the nucleotide uptake rate (Figure 2C), BODIPY-GTPγS binds to the mutant at a nearly 100× faster rate compared to the rate of BODIPY-GTPγS uptake of the wild-type. This result is comparable to several other pathogenic mutants of Gαo, such as G203R, R209C/H, E246K, and others [12,13,30]. Further, we used a hydrolyzable GTP analog labeled with BODIPY (BODIPY-GTP), allowing us to assess the rise of the transient GTP-bound form and its subsequent decline as the GTP analog is hydrolyzed, allowing us to evaluate the hydrolysis rate (Figure 2D), which reliably reflects the hydrolysis rate of the non-labeled GTP [20,31]. Surprisingly, and in opposition to many other pathogenic Gαo mutants [12,13], His_6_-Gαo[P170R] did not reveal a radical impairment of the BODIPY-GTP hydrolysis rate; the quantification demonstrated a ca. 4-fold lower *k_hydr_* than that of the wild-type His_6_-Gαo protein (Figure 2E). For the previously studied Gαo mutants, the strongly enhanced GTP uptake rate was accompanied by the almost complete loss of the ability to hydrolyze GTP, both features thought to result from conformational perturbations of the mutant proteins [12,13]. Thus, Gαo[P170R] is unique among the pathogenic mutants in the decoupling of the GTP uptake defect from the loss of the nucleotide hydrolysis.

### 3.3. Zn^2+^ Ions Elicit a Unique Effect on P170R, Kinetically Promoting It to Lose Bound GTP

**Figure 3:**
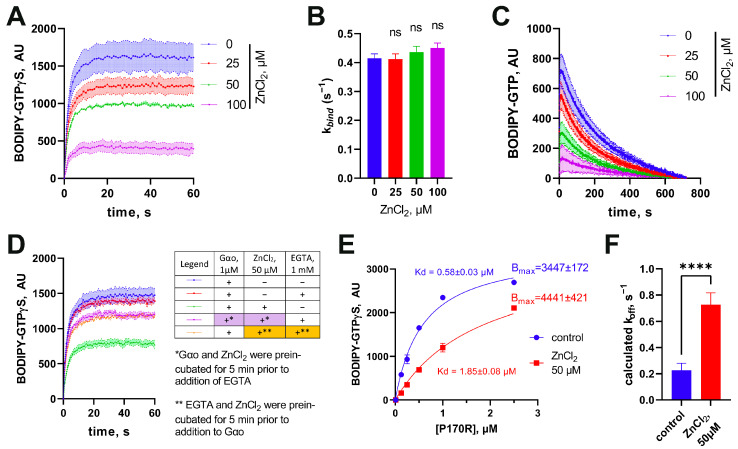
Unusual effect of zinc on His_6_-Gαo[P170R]. (**A**) Effect of progressively increasing concentrations of ZnCl_2_ on the binding rate of BODIPY-GTPγS with (**B**) quantitative analysis of the rate constants reveals that the compound affects only the fluorescence plateau, but not the binding rate. Statistical comparison was performed by 1-way ANOVA, no significance was identified using multiple comparisons. (**C**) ZnCl_2_ also has a comparable impact in the BODIPY-GTP binding assay. (**D**) The ability of ZnCl_2_ to decrease the fluorescence plateau levels is eliminated via EGTA, equally effective when added before or after the preincubation of Gαo with ZnCl_2_. (**E**) Titration of BODIPY-GTPγS with increasing concentrations of His_6_-Gαo in the absence or presence of ZnCl_2_ shows that the same maximal fluorescence plateau levels can be reached as indicated in the calculated B_max_ values, but that Zn^2+^ decreases 3-fold the dissociation constant *K_d_*, as evaluated following the formula y = B_max_*x/(*K_d_* + x). (**F**) The k_off_ value, calculated using the formula *K_d_* = k_off_/k_bind_, increases 3.5-fold in the presence of 50 µM ZnCl_2_. Data in (**E**,**F**) are mean ± SEM (n = 6); statistical significance in (**F**) is determined using *t*-test (“ns” nonsignificant; **** *p*-value < 0.0001).

Earlier, we demonstrated that the near-loss in the GTP hydrolysis in certain pathogenic Gαo mutants can be restored in the presence of Zn^2+^, which replaces Mg^2+^ in the binding site of the protein [12,17]. This effect was extremely selective towards the pathogenic mutants, with no changes in the nucleotide binding or hydrolysis experiments performed with the wild-type protein. Additionally, the presence of Zn^2+^ did not appreciably alter the binding of the non-hydrolyzable BODIPY-GTPγS to the mutants previously studied [12,17]. It was thus surprising to find out that the P170R mutant reveals yet another biochemical novelty among the pathogenic mutants, this time in relation to its response to Zn^2+^. In particular, we observed a strong concentration-dependent decrease in the maximal fluorescence achieved in the BODIPY-GTPγS assay to be induced by Zn^2+^, while the *k_bind_* kinetic constant stayed unchanged (Figure 3A,B). A similar stark drop in the peak fluorescence values as induced by Zn^2+^ was also seen in the BODIPY-GTP assay (Figure 3C).

The observed effect could result from one of the following three possible reactions of the mutant protein to Zn^2+^: (i) irreversible denaturation of the protein (which decreases the amount of molecules competent to bind guanine nucleotides leading to decreased fluorescence levels); (ii) modified conformation of the protein (that is translated into the altered environment of the bound nucleotide, and thus decreases the fluorescence yield of BODIPY); and (iii) variations in the protein’s affinity to nucleotides (BODIPY-GTP or GDP or both). To address the first hypothesis, we used 1 mM EGTA to sequester Zn^2+^ either before adding 50 μM ZnCl_2_ to His_6_-Gαo[P170R] or after 5 min of preincubating ZnCl_2_ with the protein. This experiment revealed that EGTA was similarly efficient in restoring the fluorescence levels when added before ZnCl_2_ or following the protein preincubation with the salt (Figure 3D). Thus, we reject hypothesis (i) of the irreversible denaturation of the protein by ions of zinc.

To address the other hypotheses, we titrated a fixed concentration of BODIPY-GTPγS (1 µM) with increasing concentrations of His_6_-Gαo[P170R] in the absence or presence of 50 µM ZnCl_2_ (Figure 3E). Analysis of the resultant curve indicates that the maximal fluorescence values reached at the plateau levels are not lower in the presence of ZnCl_2_, arguing against hypothesis (ii). Instead, we see that the apparent binding affinity of the mutant towards BODIPY-GTPγS is decreased >3-fold. The dissociation constant *Kd* of the His_6_-Gαo-nucleotide interaction determined in Figure 3E can be defined as the ratio of the kinetic constants of the direct and reverse reactions, $K_{d}=\frac{k_{off}}{k_{bind}}$. As we have determined that Zn^2+^ has no effect on the forward kinetic rate constant, *k_bind_* (Figure 3B), we conclude that the ion increases by more than 3-fold the *k_off_* kinetic constant (Figure 3F). In other words, Zn^2+^ forces the P170R mutant to spontaneously lose the bound BODIPY-GTPγS, by inference promoting the reuptake of GDP. These properties of the P170R mutant distinguish it from the other pathogenic Gαo mutants studied so far, which either responded to zinc with a restored/increased GTPase reaction (G203R, E246K, R209C) or were fully unresponsive to zinc (T241_N242insPQ) [12,17], possibly identifying a third biochemically defined category of the pathogenic mutations.

### 3.4. The P170R Substitution Perturbs the Protein’s Structure, Stability, Cellular Interactions and Localization

**Figure 4:**
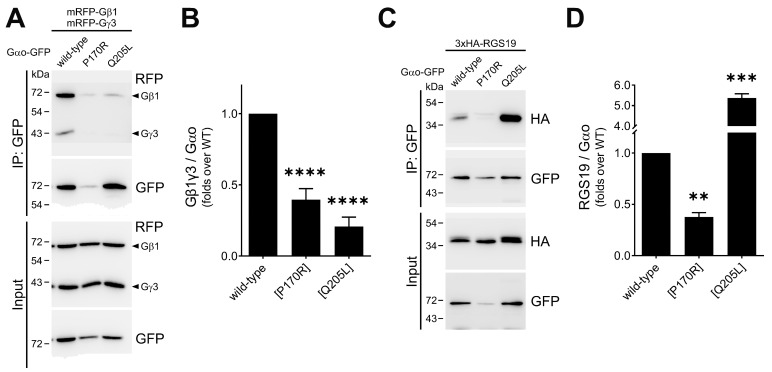
Reduced cellular interactions of Gαo[P170R]. (**A**,**B**) The P170R mutant tagged internally with GFP at Gly92 demonstrates poor interaction with RFP-labeled Gβγ (mRFP-tagged β1 and γ3) subunits in immunoprecipitation assays, comparable to that of the constitutively active Q205L mutant. Representative Western blots (**A**) and quantification normalized to the precipitated Gαo^Gly92^-GFP (**B**) are shown. (**C**,**D**) The interaction of Gαo[P170R]^Gly92^-GFP with 3xHA-tagged RGS19 analyzed by immunoprecipitation shows that it is halved for the mutant. Representative Western blots (**C**) and quantification normalized to the precipitated Gαo^Gly92^-GFP (**D**) are shown. Statistical comparison was performed via Student’s *t*-test, significance is shown as ** *p* < 0.01, *** *p* < 0.001, and **** *p* < 0.0001.

To analyze whether Gαo[P170R] can form the heterotrimer, we performed a co-IP-based evaluation of its interaction with Gβγ subunits. This assay uses Gαo^Gly92^-GFP variants internally tagged at the Gly92 position and mRFP-tagged Gβ1 and Gγ3, which do not obstruct heterotrimer assembly nor proper intracellular localizations [12,13,19,21]. The robust formation of the heterotrimer can be observed for the wild-type variant; the constitutively active (GTPase-dead), non-pathogenic Q205L mutant serves as a negative control unable to interact with Gβγ (mRFP-β1/mRFP-γ3, Figure 4A,B). We find that Gαo[P170R]^Gly92^-GFP is as deficient in the binding to Gβγ as the Q205L form, an observation that correlates with the fast GTP uptake of the P170R mutant, which should lead to the predominantly GTP-loaded state of the mutant in cells.

Next, we examined the interactions of the mutant and both controls with RGS19 (tagged with 3 × HA)—the partner whose interactions with pathogenic Gαo variants are typically strongly reduced [12,13,17,19]. As anticipated from these earlier analyses, we find a >2-fold reduction in the ability of the Gαo[P170R]^Gly92^-GFP mutant to interact with 3×HA-RGS19 compared to the wild type (Figure 4C,D); Gαo[Q205L]^Gly92^-GFP, as expected, shows a dramatically enhanced interaction with 3×HA-RGS19. Upon comparison with the panel of other pathogenic variants studied [12,13,17,19], it can be concluded that the loss/reduction in the ability to interact with RGS19 is a uniform feature of the *GNAO1* encephalopathy mutants, regardless of whether they are deficient in GTP hydrolysis (as, e.g., G203R, E246K, or R209C) or not (as the P170R mutant studied here).

**Figure 5:**
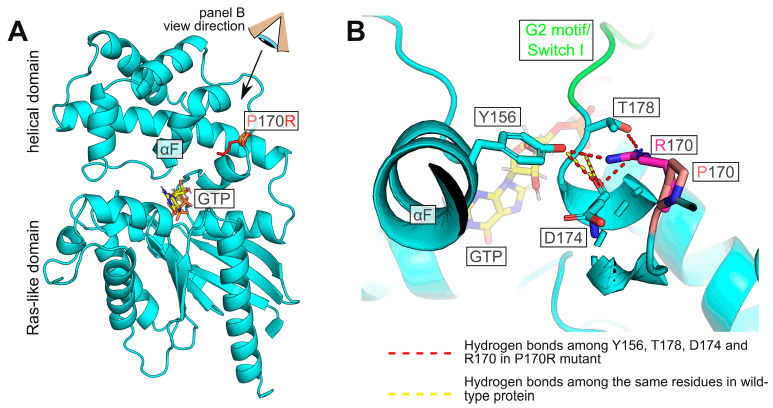
Model of human Gαo with P170R substitution. (**A**) Overview of the protein showing the mutation site with the Pro (pink) or Arg (red) side chain. (**B**) Detailed view (from the angle indicated in panel (**A**)) of the substituted position. The hydrogen bond network between the η-O atom of Y156 and the backbone oxygens of T178 and D174 is perturbed by the mutation, and Arg170 forms hydrogen bonds with the η-O of Y156 and the backbone O of D174 via one of its η-Ns. Further, the same η-N of R170 engages γ-O of T178. These perturbations are likely to affect the G2 motif (green).

To gain insight into the possible structural basis for the deficient nucleotide handling and cellular interactions of the P170R mutant, we modeled its structure (Figure 5) using SWISS MODEL [23], which performs AlphaFold-based homology modeling and energy minimization. As shown in Figure 5A, the original P170 residue is not involved in any critical interactions with neighboring residues. Somewhat surprisingly, the protein has sufficient space to accommodate the bulky Arg residue at this position without causing major steric collisions. We further find that the positively charged guanidino group of Arg in the P170R mutant is located in close proximity to residues Y156, D174, and T178, which in the wild-type protein are involved in the intricate hydrogen bond network with each other (yellow dashed lines in Figure 5B) and also with other residues in the protein. Their novel interactions with R170 lead to hydrogen bonds with the η-O of Y156 and the backbone O of D174 via one of the η-Ns of Arg, as well as an additional interaction with the side chain γ-O of T178 (red dashed lines in Figure 5B). These changes are predicted to result in the altered position and reduced flexibility of the adjacent G2 motif/Switch I region (green in Figure 5B). These effects provide a likely explanation for the altered nucleotide binding and hydrolysis behavior, as the G2 motif residues are known to bind guanine nucleotides and participate in the GTPase activity [32]. In addition, this motif is one of the regions that undergoes the most significant conformational changes upon nucleotide exchange and mediates interactions with downstream partners, particularly with RGS proteins [33].

**Figure 6:**
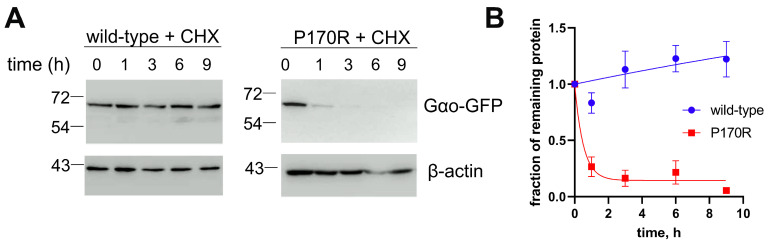
Reduced cellular stability of Gαo[P170R]^Gly92^-GFP. (**A**) Using cycloheximide (CHX) to block de novo protein synthesis, we find that Gαo^Gly92^-GFP wild-type is stable at least during the 9 h of the experiment (**A**), while P170R is rapidly degraded. Representative Western blots are shown. β-actin is used as a control. (**B**) Quantification from 3 biological replicates shown as mean ± SEM displays the half-life of P170R as 0.4 h.

In our pull-down experiments (Figure 4A,C), we find that the quantities of Gαo[P170R]^Gly92^-GFP that are detected after the completion of the immunoprecipitation protocol are strongly reduced compared to the similarly tagged wild-type or Q205L proteins. This loss in the P170R variant may suggest its reduced stability. In agreement, Gαo[P170R]^Gly92^-GFP’ cellular expression levels are reduced compared to those of Gαo wild-type or Q205L (Figure 4A,C, input panels), similar to many other pathogenic Gαo variants; it is noteworthy that similar reductions in the expression levels are seen for the pathogenic variants when they are expressed non-tagged or with the internal GFP-tag [13]. To directly assess the stability of Gαo[P170R]^Gly92^-GFP, we used cycloheximide to block de novo protein synthesis, monitoring the decay time of internally GFP-tagged Gαo, wild-type, and P170R. Remarkably, we find that while the wild-type Gαo^Gly92^-GFP variant is stable over the 9 h duration of the cycloheximide experiment, Gαo[P170R]^Gly92^-GFP is rapidly degraded (Figure 6A), with the half-life of less than 1 h (Figure 6B).

**Figure 7:**
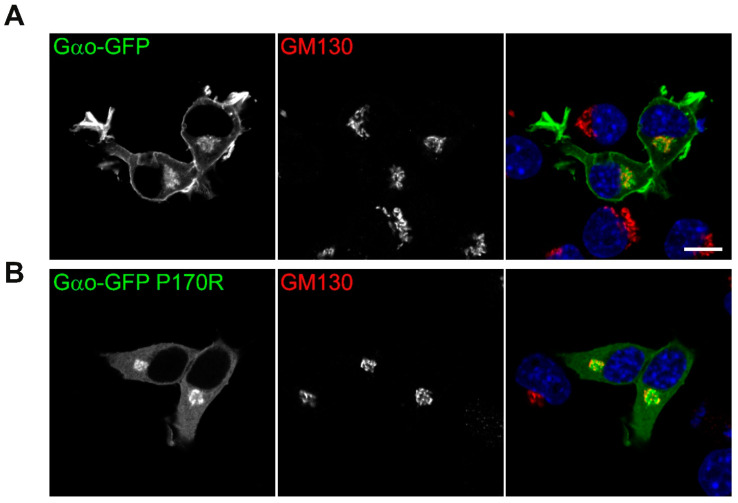
Aberrant intracellular localization of Gαo[P170R]^Gly92^-GFP. Confocal images of intracellular localization of (**A**) wild-type and (**B**) P170R mutant Gαo^Gly92^-GFP cotransfected with RFP-labeled Golgi marker GM130 in N2a cells. DAPI staining labels nuclei in blue. Plasma membrane localization is clearly visible for the wild-type protein while starkly disrupted for the P170R mutant; the Golgi pool of the mutant protein remains unperturbed. Scale bar: 10 µm.

Finally, we addressed one of Gαo’s most important characteristics: its localization within the cells. When compared to wild-type Gαo^Gly92^-GFP that distinctly localizes to the plasma membrane and the Golgi apparatus [21,34] (Figure 7A), the P170R mutant shifts its plasma membrane localization to a diffuse cytoplasmic pattern while maintaining the Golgi localization (Figure 7B). We previously discovered that the loss/reduction in the plasma membrane localization of a pathogenic Gαo variant correlates with the epileptic manifestations it clinically induces [13]. In this regard, the P170R mutation reliably follows this pattern.

## 4. Discussion

Dominant de novo mutations in *GNAO1* underlie the severe pediatric encephalopathy with motor dysfunction, epilepsy, and developmental delay. Mostly producing Gαo variants with single amino acid substitutions, these mutations affect both prenatal development of the nervous system and brain functioning after birth [10,11]. While the nature of these dominant mutations (gain-of-function vs. dominant negative) has been debated, we have recently proposed a unifying mechanism of the molecular etiology of the disease, with the neomorphic (gaining novel molecular functions) nature of the mutations driving *GNAO1* encephalopathy [12,13,35]. Earlier studies of the previously identified Gαo mutants broadly characterize them as deficient in guanine nucleotide handling, intracellular localization, cellular interactions, and signaling [12,13,17,19,30,36].

Here, we discover a novel c.509C>G de novo mutation in *GNAO1* in two unrelated pediatric patients suffering from developmental and epileptic encephalopathy. The resultant Gαo[P170R] mutant variant displays a hyper-accelerated GTP binding rate, accompanied by a mildly decreased rate of GTP hydrolysis. As may be expected for a mutant that constitutively resides in the GTP-bound state, P170R shows low binding to Gβγ subunits. This constitutive GTP binding, however, is not reflected in the stimulated interaction with RGS19, which is instead reduced. This feature has been uniformly observed for many other pathogenic *GNAO1* variants and likely reflects the inability of the Gαo mutants to adopt the properly activated conformation upon loading with GTP [12,13]. Like other mutants with severely disrupted Gβγ binding [19], P170R has disrupted plasma membrane localization (while retaining its Golgi occupancy), which agrees with the presence of epileptic manifestations in the affected patients [13]. The structural modeling of the mutant suggests that the P170R substitution impacts the Switch I region, which is important for nucleotide handling and cellular interactions.

Our assays were performed using the tagged version of Gαo: His_6_-Gαo for the in vitro biochemical assay and Gαo^Gly92^-GFP for the cellular assay. Recombinant His_6_-Gαo is active with normal protein–protein interactions and, importantly, shows comparable kinetic profiles to native Gαo purified from the bovine brain [12,13,17,19,20,21,27,28]. In this present study, the calculated BODIPY-GTPγS binding rate (*k*_bind_) of wild-type His_6_-Gαo was 0.228 min^−1^, whereas the binding rate of [^35^S]GTPγS of bovine brain Gαo was 0.21 min^−1^ [37]. This agreement also confirms that the BODIPY moiety has little influence on the kinetic profile of either recombinant or native Gαo. In cellular assays, internal tagging of Gα subunits with various tags (GFP, CFP, Renilla luciferase, NanoLuc) has been widely used in the GPCR field, with the insertion of the tag after the Gly92 in Gαo being optimal for various GPCR biosensors [38,39,40]. In another study where we evaluated the molecular defects of 16 pathogenic Gαo variants, there is a highly significant correlation in Gβγ interaction with mutant Gαo determined using two independent methods: (i) co-IP using Gαo^Gly92^-GFP and (ii) Gβγ displacement in BRET assay using non-tagged Gαo [13]. Further, RGS19 interaction with non-tagged Gαo mutants also recapitulates the results using Gαo^Gly92^-GFP (manuscript in preparation). These findings confirm that the GFP tag does not affect the Gαo interaction with cellular binding partners.

Currently, there exists no efficient treatment for *GNAO1* encephalopathy, apart from the limited efficiency of deep-brain stimulation that represents a symptomatic (but often life-saving) treatment for the severe episodes of motor dysfunction, without affecting the other disease manifestations such as epilepsy or developmental delay [3,26]. Thus, the search for novel treatments is urgently needed, and the repositioning of approved drugs for the novel indication provides a sensible avenue for a rare disease (as opposed to, e.g., de novo drug discovery and development) [35,41]. In this quest, caffeine and salts of zinc have emerged as candidate treatments. While the effects of caffeine may be mediated by antagonism on adenosine receptors to counterbalance the deficient Gαo-mediated signaling [42,43], ions of zinc have been shown to go to the very core of the pathogenic mutants’ Gαo activities, restoring the lost GTP hydrolysis of the mutants and their cellular interactions [12,35]. In a *Drosophila* model of the disease, dietary zinc supplementation was able to partially restore the deficient motor activities and life span [12]. Given the fact that dietary zinc has been approved for the treatment of a number of diseases, and the safety profile of this drug is well known [15,16], we hope to move ahead with a formal clinical trial in patients with a subset of *GNAO1* mutations, following the doses approved for the treatment of the pediatric Wilson disease [15].

These considerations drove us to investigate the responsiveness of the P170R mutation to zinc salts. We found that the biochemical and cellular aberrations of Gαo[P170R] are enforced with its so far unique, among the pathogenic variants, response to Zn^2+^ ions. Instead of the stimulation of GTP hydrolysis via Zn^2+^ observed for one category of Gαo mutants [12] or no effect for another category of the mutants [17], zinc ions strongly reduce the Gαo[P170R]’s affinity for GTP. This effect is not due to the alteration of the forward kinetic rate constant but, instead, to the stimulated *k_off_* constant. In other words, Zn^2+^ stimulates the loss in GTP by the GTP-bound Gαo[P170R], defining this variant as a distinct biochemical category within the pathogenic mutants. Despite this novel mechanism of action, we may anticipate that zinc supplementation could lead to a decrease in the GTP-bound form of the mutant at the organism/patient level—similarly to the E246K, G203R, or R209C forms, where zinc restores the GTPase activity of the mutants [12]. In case it is the GTP-bound form of Gαo[P170R] that is the major driver of the disease progression, the Zn^2+^ regimens could become a viable treatment option for the disease driven by the P170R mutation. However, given the new biochemical mode of action of zinc towards this mutant, distinct from that we previously validated as beneficial in the animal model of the disease [12], we think it useful that an animal model of P170R-driven encephalopathy be established, and the positive action of zinc supplementation in this model be demonstrated, prior to the recommendation of such treatment in patients.

## Data Availability

The data presented in this study are fully disclosed in the main article and its Supplementary Materials.

## Supplementary Materials

The following supporting information can be downloaded at: https://www.mdpi.com/article/10.3390/cells12202469/s1, References [44,45,46,47] are cited in the Supplementary Materials.
