# Supplementary material for: Clinical Cases and the Molecular Profiling of a Novel Childhood Encephalopathy-Causing GNAO1 Mutation P170R
Source: Cells. 2023 Oct 17;12(20):2469. doi: 10.3390/cells12202469 (PMC10605901; doi:10.3390/cells12202469)
Supplement: Supplementary file 1 [file cells-12-02469-s001.zip › cells-2531245-supplementary.pdf]

SUPPLEMENTARY MATERIALS AND METHODS

Recombinant His_6-Gαo

His_6-tagged Gαo (wild-type and mutant P170R) proteins were expressed in *Escherichia coli* Rosetta(DE3)pLysS (Novagen, 70956) as previously described [12, 20]. Briefly, transformed bacteria were grown at 37°C to an OD_{600} of 0.6 and cooled to 18°C for at least 30 min before induction with 0.25 mM isopropyl-β-D-thiogalactopyranoside. After additional growth overnight at 18°C, bacteria were harvested by centrifugation 3,500xg at 4°C and resuspended in TBS (20 mM Tris-HCl (pH 7.5) and 150 mM NaCl) supplemented with 1 mM PMSF and 30 mM imidazole (all from Sigma-Aldrich). Cells were disrupted with a high pressure cell press homogenizer and extracts were cleared by centrifugation at 15,000xg for 15 min at 4°C. Supernatants were incubated overnight with Ni-NTA Agarose beads (QIAGEN) on a rotary shaker at 4°C. Beads were washed thrice with 10 resin volumes of ice-cold wash buffer (TBS supplemented with 10 mM imidazole) and bound proteins were GDP-loaded in TBS supplemented with 3% glycerol, 10 mM $MgCl_2$, 0.1 mM DTT, and 200 μM GDP. The beads were washed three more times with at least 10 resin volumes of wash buffer and finally eluted with TBS containing 300 mM imidazole. Imidazole was removed by buffer exchange to TBS using Vivaspin Centrifugal concentrators. Protein concentration was measured using the Bradford assay, and the purity was analyzed by SDS-PAGE followed by Coomassie staining.

BODIPY- GTPγS and BODIPY-GTP as tools to determine GTP uptake and hydrolysis rate of Gαo

BODIPY-GTPγS was used to measure the GTP uptake rate (k_{bind}) of His_6-Gαo. GTPγS as a non-hydrolyzable GTP analog, is routinely used to determine GTP binding rates, as the time course of the association reaction between GTPyS and Gαo is very similar to that of the dissociation of bound GDP, the rate-limiting step in guanine nucleotide exchange [44]. Previous studies have shown that the fluorescence increase of BODIPY-GTPγS upon addition of His_6-Gαo has a kinetic profile similar to that of GDP release [44, 45] – a rate-limiting step in nucleotide exchange - indicating the suitability of BODIPY-GTPγS as a tool to determine the GTP binding rate of Gα subunits.

BODIPY-GTP was used to measure the GTP hydrolysis rate (k_{hydr}) of His_6-Gαo. A previous study reported that the basal GTPase activity of Gαo and its modulation by GAPs and a synthetic peptide GAP inhibitor, exhibited similar profile between BODIPY-GTP and that reported for GTP [31].

Calculation of GTP uptake and hydrolysis rates

To calculate k_{bind}, BODIPY-GTPγS fluorescence increase upon binding to His_6-Gαo was normalized (plateau= 100%) to better observe the different binding rates between the wild-type and mutant Gαo. k_{bind} was then calculated using one-phase exponential equation in GraphPad Prism:

Y=Y0 + (Plateau-Y0)*(1-exp(-K*x)); with K as k_{bind} (sec^{-1}).

To calculate k_{hydr}, BODIPY-GTP fluorescence increase upon binding to His_6-Gαo was baseline-adjusted by subtraction of the mean fluorescence value at the end of the curve (indicating complete GTP hydrolysis and thus return to baseline). Calculation of k_{hydr} was then performed using exponential equation for intermediate product of two-step irreversible reaction using GraphPad Prism.

To model the kinetics of BODIPY-GTP uptake and hydrolysis by Gαo, the processes of BODIPY-GTP uptake and hydrolysis were modeled as follows [46]:

$$\text{G}\alpha\text{o-GDP} + \text{BODIPY-GTP} \xrightarrow{V1} \text{G}\alpha\text{o-BODIPY-GTP} \xrightarrow{V2} \text{G}\alpha\text{o-GDP} + \text{BODIPY-Pi},$$

$$\text{or } n1 + n2 \xrightarrow{V1} n3 \xrightarrow{V2} n1 + n4,$$

where V1 is the rate of BODIPY-GTP uptake, and V2 – hydrolysis by Gαo, and can be described as follows:
V1 = k1×n1, and V2 = k2×n3,

where k1 is the BODIPY-GTP association rate constant (numerically identical to k_{bind} derived from BODIPY- GTPγS assay), and k2 is the hydrolysis rate constant.

From this it follows that
d[Gαo-BODIPY-GTP] / dt = dn3 / dt = V1 - V2 = k1×n1 - k2×n3.

Since [Gαo-GDP] + [Gαo-BODIPY-GTP] = M = const, where M is the total concentration of Gαo, the previous equation can be rewritten as:
dn3 / dt = k1 (M - n3) - k2 n3

The differential eq. 1 was then solved using the master equation below [47] and the data were fitted using GraphPad Prism.
$n3 = (n1\ k1 / (k2 - k1))\ (e^{-k_1 t} - e^{-k_2 t})$

Analysis of GTP uptake and hydrolysis kinetic profiles of His_6-Gαo[P170R] in the presence of $ZnCl_2$ and EGTA
To evaluate the effect of $ZnCl_2$ on the P170R mutant, $ZnCl_2$ (25-100 µM) was mixed with His_6-Gαo[P170R] (1 µM) and was incubated for 5 minutes before being subjected to GTP binding/hydrolysis assays.

In the assay using EGTA, EGTA (1 mM) was added in two different ways:

(i) Gαo[P170R] was pre-incubated with $ZnCl_2$ (50 µM) for 5 minutes to ensure that Zn^{2+} had interacted with Gαo, then EGTA was added just before measurement with BODIPY-GTPγS
(ii) $ZnCl_2$ was pre-incubated with EGTA for 5 minutes to ensure that Zn^{2+} had been chelated by EGTA, then Gαo was added just before measurement with BODIPY-GTPγS

Determination of BODIPY-GTPγS affinity to His_6-Gαo[P170R] in the presence of $ZnCl_2$
Determination of BODIPY-GTPγS affinity for His_6-Gαo[P170R] was performed by titrating BODIPY-GTPγS with increasing concentration of His_6-Gαo[P170R]. His_6-Gαo[P170R] (0.5-2.5 µM) were pre-incubated with $ZnCl_2$ (50 µM) for 5 minutes before the addition of BODIPY-GTPγS (1 µM). The fluorescence increase was measured for 60 sec (when equilibrium has been reached as in Fig. 3A), and this fluorescence increase was plotted on the Y-axis as the specific complex formation of BODIPY-GTPγS and His_6-Gαo[P170R]. These data were then fitted to the equation y = Bmax*x/(Kd + x) to calculate the apparent K_d.

Alternatively, K_d is the ratio between the kinetic constants of the direct and reverse reactions: $K_d=k_{off}/k_{bind}$, where we used this equation to calculate the k_{off} of BODIPY-GTPγS as depicted in Fig.3F.

In fact, the more common variant of the assay used to determine K_d is to vary the small molecule rather than the protein concentration. However, K_d is an equilibrium constant derived for the reversible binding of two ligands of any type. Therefore, there is no computational difference between using either of these ligands at different concentrations. In this particular assay, variation in the concentration of the fluorescent ligand would mean that the fluorescence maximum at each concentration would also increase, which must be taken into account at least computationally, but better experimentally as it may be non-linear due to a number of factors. This would make such an assay technically cumbersome, which is why we have varied the protein concentration in this case.

Molecular modeling of Gαo[P170R]

Molecular modeling of Gαo[P170R]

To obtain the molecular model of the mutant Gαo in the energy-minimized state, we used the integrated SWISS-MODEL server. The server provides an automated pipeline that includes the following steps:

1) Homology modeling using the ProMod3/OpenStructure algorithm (using Gαi1, PDB ID 1GIL as the main source of homology information).
2) Global optimization of rotamers using TreePack with SCWRL4 energy minimization
3) Refined energy minimization using OpenMM and CHARMM22/CMAP force field.

The ligand position is transferred from the template provided that the major coordinating residues are preserved. Given the high degree of homology and residue conservation between G proteins and the functional similarity between the mutants, we therefore expect that such modeling will produce an overall accurate representation of a crystal structure of a wild-type and mutant Gαo protein, provided that they were crystallized under the same conditions as the template Gαi1 protein.
